# Understanding the Role of Yeast Yme1 in Mitochondrial Function Using Biochemical and Proteomics Analyses

**DOI:** 10.3390/ijms232213694

**Published:** 2022-11-08

**Authors:** Kwan Ting Kan, Michael G. Nelson, Chris M. Grant, Simon J. Hubbard, Hui Lu

**Affiliations:** School of Biological Sciences, Faculty of Biology, Medicine and Health, Manchester Academic Health Science Centre, The University of Manchester, Manchester M13 9PT, UK

**Keywords:** AAA proteinase, mitochondrial function, mitochondrial proteomics, OXPHOS complex

## Abstract

Mitochondrial i-AAA proteinase Yme1 is a multifunctional protein that plays important roles in maintaining mitochondrial protein homeostasis and regulating biogenesis and function of mitochondrial proteins. However, due to the complex interplay of mitochondria and the multifunctional nature of Yme1, how Yme1 affects mitochondrial function and protein homeostasis is still poorly understood. In this study, we investigated how *YME1* deletion affects yeast *Saccharomyces cerevisiae* growth, chronological life span, mitochondrial protein homeostasis and function, with a focus on the mitochondrial oxidative phosphorylation (OXPHOS) complexes. Our results show that whilst the *YME1* deleted cells grow poorly under respiratory conditions, they grow similar to wild-type yeast under fermentative conditions. However, the chronological life span is impaired, indicating that Yme1 plays a key role in longevity. Using highly enriched mitochondrial extract and proteomic analysis, we show that the abundances of many mitochondrial proteins are altered by *YME1* deletion. Several components of the respiratory chain complexes II, III, IV and V were significantly decreased, suggesting that Yme1 plays an important role in maintaining the level and function of complexes II-V. This result was confirmed using blue native-PAGE and in-solution-based enzyme activity assays. Taken together, this study shows that Yme1 plays an important role in the chronological life span and mitochondrial protein homeostasis and has deciphered its function in maintaining the activity of mitochondrial OXPHOS complexes.

## 1. Introduction

Mitochondria are critically important organelles of eukaryotic cells, which regulate a wide range of cellular functions, from cell survival and growth to cell death. In particular, mitochondrial respiration and the TCA cycle generate a large amount of ATP and various important metabolites, allowing mitochondria to integrate many essential activities in eukaryotic cells. Moreover, the activity of the mitochondrial electron transport chain (ETC) requires the careful coordination of import and assembly of proteins synthesised in the cytosol that must be assembled in the mitochondrial matrix at correct subunit stoichiometrys [1,2]. Thus, mitochondrial biogenesis and function are highly regulated, and the mitochondrial proteome undergoes constant resculpting in response to the changing demands of cells [3,4]. Proteolytic breakdown of damaged and unwanted proteins is a major strategy for mitochondrial protein quality control, and the AAA (ATPases Associated with diverse cellular Activities) proteases provide a major route for protein degradation in mitochondria. Not surprisingly, mutations in these proteases cause mitochondrial dysfunction and lead to diverse neurodegenerative disorders in humans [5,6,7,8].

Mitochondria have two highly conserved inner membrane-bound AAA proteinase complexes: called i-AAA and m-AAA, as their functional domains are located in the mitochondrial intermembrane space (i-AAA) and matrix (m-AAA), respectively. Studies have shown that they play important roles in maintaining mitochondrial proteostasis by facilitating the assembly of respiratory complexes, preventing accumulation of potentially harmful, damaged and/or unwanted proteins, as well as regulating protein functions via peptide cleavage to activate or inactivate target proteins [9,10,11]. Yme1 is the sole subunit protein of the i-AAA proteinase complex in the yeast *Saccharomyces cerevisiae* and is a multifunctional protein involved in mitochondrial protein turnover, protein import, folding and maturation. For example, Yme1 supports the quality control of proteins located in the inner membrane, outer membrane and the intermembrane space (IMS) [12,13,14,15]. It serves as a non-conventional translocation motor during import of PNPase into the IMS [16]. Yme1 also mediates lipid synthesis [17], maintenance of mitochondrial morphology [18] and plays an essential role in yeast mitophagy [19,20]. The importance of i-AAA has also been shown by the finding that knockdown of the human homologue *YME1L* in embryonic cells impairs cell proliferation and sensitises cells to apoptosis [21]. However, despite many studies on i-AAA, how *YME1* deletion affects mitochondrial function and cell growth is still not fully understood. For example, Yme1 plays important roles in maintaining the electron transport chain (ETC) function, yet how Yme1 affects the component proteins of ETC is unknown and the limited experimental results are seemingly contrasting. This emphasises the lack of a clear understanding of Yme1′s role in ETC function. More studies at the cellular and molecular levels are required to understand the function of Yme1.

**Figure 1:**
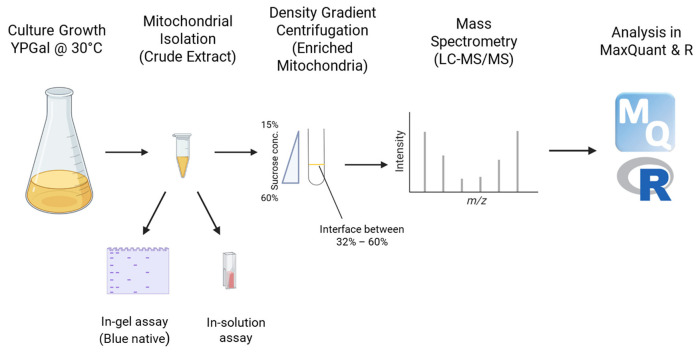
Workflow for mitochondria isolation, activity and proteomics analyses for the WT and *Δyme1* samples.

In this current study, we investigated how *YME1* deletion (*Δyme1*) affects yeast cell growth, chronological life span, mitochondrial protein homeostasis and function, with a focus on the mitochondrial oxidative phosphorylation (OXPHOS) complexes. Our results show that whilst the *YME1* deleted strain grows poorly under respiratory conditions, it is unaffected in growth under fermentative conditions. However, the chronological life span of the mutant is decreased even under fermentative conditions. Furthermore, we enriched mitochondria from cells grown in YPGal and investigated how *YME1* deletion affects mitochondrial protein homeostasis with mass spectrometry-based approach using the workflow illustrated in Figure 1. The yeast proteome was studied extensively in past studies using mass spectrometry, whether with the whole cell [22,23] or enriched mitochondria [12,24]. In this study, mass spectrometry-based proteomics analysis showed that the abundances of many mitochondrial proteins are altered in the absence of Yme1. In particular, the abundance of several components of the respiration chain complexes and TCA cycle are decreased. Furthermore, using blue native-PAGE-based activity assays, we confirmed that *YME1* deletion heavily impairs the mitochondrial respiration chain complexes II, III, IV and V activity. Taken together, our data show that Yme1 plays an important role in the chronological life span of yeast due to its role in maintaining mitochondrial protein homeostasis, including the abundance of several subunits and thus the activity of OXPHOS complexes.

## 2. Results

### 2.1. Effect on YME1 Deletion on Yeast Grow under Fermentative and Respiratory Conditions

**Figure 2:**
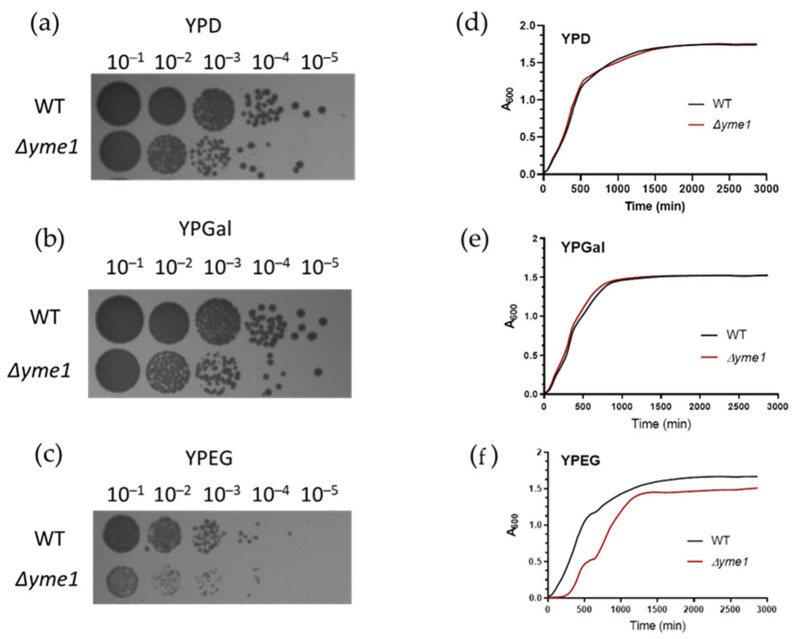
Effect of *YME1* deletion on yeast growth. (**a**–**c**) Spot test analysis of the WT and *YME1* deletion strains on different growth media as indicated. Log phase cells were spotted on fermentable media YPD (**a**), YPGal (**b**) and non-fermentable medium YPEG (**c**) plates at various dilutions, followed by incubation at 30 °C for 3 days before taking pictures. (**d**–**f**) Cell growth of the WT and *YME1* deletion cells in liquid YPD (**d**), YPGal (**e**) and YPEG (**f**), respectively, at 30 °C, time courses of optical intensity change at 600 nm was measured.

First, the effect of *YME1* deletion (*Δyme1*) on yeast growth under fermentative (YPD, YPGal) and respiration (YPEG) conditions was examined. The spot test experiment showed *YME1* deletion did affect cell growth under fermentative conditions compared with the wild-type strain (Figure 2a,b). The effect is more severe when cells were grown under respiratory conditions (Figure 2c). The growth curve showed the *Δyme1* strain grown under fermentative conditions had a similar growth profile compared with the wild-type strain (Figure 2d,e). However, under YPEG respiratory conditions, growth of the *Δyme1* strain was impaired (Figure 2), the *Δyme1* strain grew much slower than the WT yeast with an increased doubling time (~2.8 h) compared with that of the WT cells (1.6 h). This is consistent with previous results that *YME1* deletion impairs yeast grow under respiratory conditions, and Yme1 plays an important role in mitochondrial function [21]. Interestingly, at around 600 min, a second increase can be observed in the growth curve for *Δyme1*. The reason was unclear and it was speculated that this might be due to a metabolism switch.

**Figure 3:**
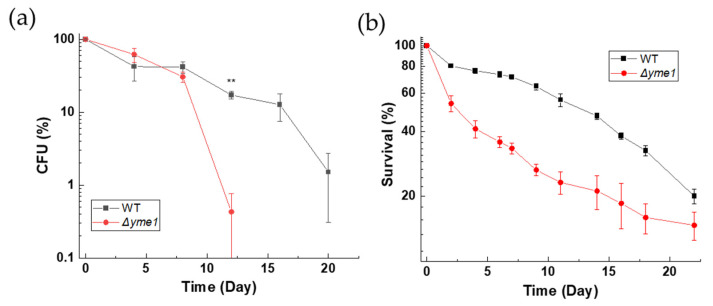
Effects of YME1 deletion on chronological life span of yeast cells. (**a**) Chronological life span (CLS) of yeast measured using a colony forming unit (CFU) count. WT and *Δyme1* cells were inoculated into YPGal at 30 °C for 2 days to reach stationary phase (day 0). Subsequently, aliquots of OD_600_ = 0.1 cell suspension were spread on YPGal plates in the days indicated. Plates were incubated at 30 °C for 2 days prior CFU count. Data represent percentage of CFU compared with day 0. (**b**) Chronological life span of yeast cells measured by flow cytometry coupled with propidium iodide staining. Cells were first grown in SCD media supplemented with 4-fold excess of uracil, leucine, tryptophan, adenine and histidine to reach stationary phase (day 0) before viability was assessed by PI staining and FCM analysis for three independent experiments. Data represent percentage of live cells relative to day 0 with relation to time. All error bars represent standard error of mean (SEM) of at least three independent experiments of two different cell cultures (n = 6). The statistic was done using Student’s t test, ** *p* < 0.01.

We reasoned that any effect of YME1 deletion on cells under fermentative growth conditions was weak and could have an accumulative effect on cell survival and viability during longer growth periods. Therefore, we next investigated how *Δyme1* affects the chronological life span (CLS) of yeast. Consistent with our prediction, colony formation analysis (Figure 3a) showed that the viability of the *Δyme1* strain started to decrease after 8 days. There was a statistically significant difference (*p* < 0.01) between WT and *Δyme1* at day 12, and no colonies were observed for the mutant at days 16 and 20 (Figure 3a). In addition, cell viability was also measured using flow cytometry coupled with the use of propidium iodide (PI) dye (Figure 3b) which acted as a dead cell reporter [24]. In this case, a synthetic fermentation medium (SCD+) was used to allow better control of the composition of nutrients in the medium. The result was in agreement with that of the CFU analysis, showing that YME1 deletion decreases the CLS of yeast.

### 2.2. Effects of YME1 Deletion on Mitochondrial Membrane Potential and Cardiolipin Content

**Figure 4:**
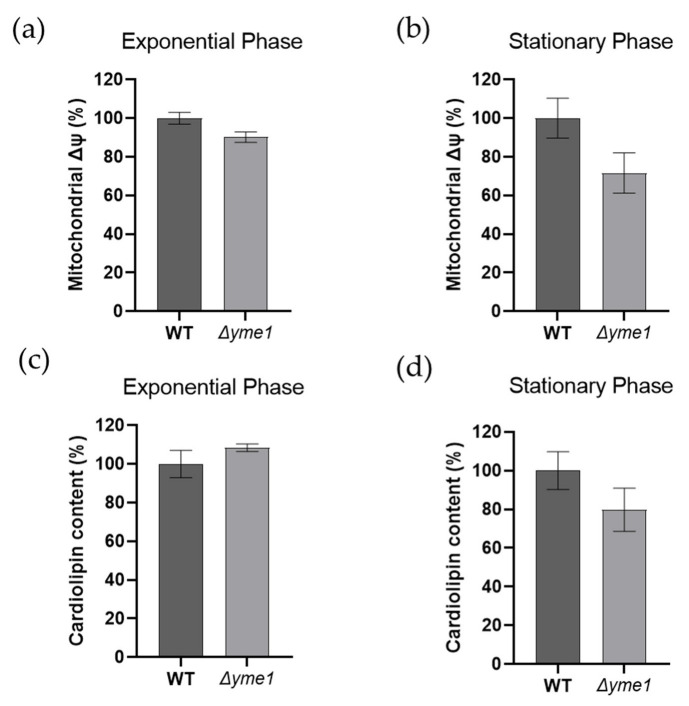
Effects of YME1 deletion on yeast mitochondrial membrane potential (Δψ) and cardiolipin content. (**a**,**b**) Mitochondrial membrane potential (Δψ) of yeast cells grown in YPGal at exponential (**a**) and stationary (**b**) phases were measured using fluorescence dye DiOC_6_. (**c**,**d**) Cardiolipin content of the yeast cells grown in YPGal at exponential (**c**) and stationary (**d**) phases were analysed using flow cytometry coupled with fluorescent dye NAO. For both assays, cells equivalent to OD_600_ = 1 were harvested and incubated with the specific dye at 30 °C for 30 min prior to analysis by flow cytometry. The error bars represent standard error of mean (SEM) of three independent experiments of three different cell cultures (n = 9). The statistic was done using Student’s t test, all results show no significant difference with *p* > 0.05.

It is well known that both mitochondrial membrane potential (Δψ) and cardiolipin play important roles in energy production in mitochondria. Thus, we measured how *YME1* deletion affects both factors using flow cytometry coupled with specific dyes, namely DiOC_6_ (for membrane potential) and NAO (for cardiolipin), respectively (Figure 4). The results showed no significant differences (*p* > 0.05) in mitochondrial membrane potential (Figure 4a,b) or cardiolipin content (Figure 4c,d) between the WT and mutant strains and during both the exponential growth and early stationary phases. There was a slight decrease in the membrane potential during the exponential phase, but the difference was not significant due to large experimental errors (Figure 4b).

### 2.3. Mitochondrial Proteomics Analysis of YME1 Deletion Reveals Defects in Mitochondrial Function under Fermentative Condition

To understand how *Δyme1* affects mitochondrial protein homeostasis as a whole, we took advantage of the fact that the *Δyme1* strain grows similarly to the WT under fermentative growth conditions and purified mitochondria from the WT and *YME1* deletion yeast strains grown in YPGal (see Method for detail). Here, galactose (Gal) was used as a carbon source instead of glucose since cells grown in galactose have more mitochondria and increased activity of respiratory enzymes compared with glucose-grown cells [25]. Following isolation of mitochondria and protein extraction, proteomics analysis was performed using a label-free mass spectrometry (MS) proteomics approach. The raw data from mass spectrometry were processed with MaxQuant [26] and MSstat package in R [27] to identify and quantify proteins that display significantly changes in abundance, either up or down. Proteins displaying significant changes in the mutant strain were investigated for significant functional enrichment using the Gene Oncology Resource [28,29].

**Figure 5:**
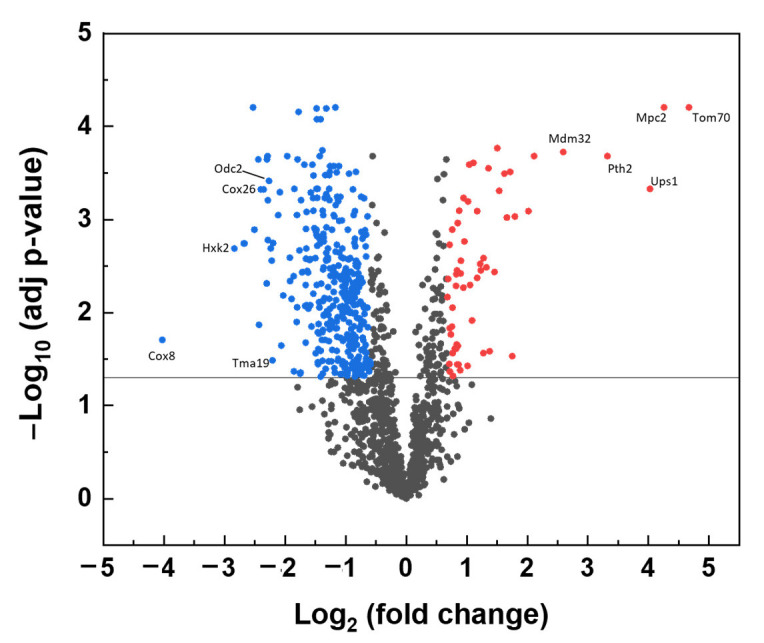
Volcano plot of effect of *Δyme1* on mitochondrial protein abundance compared with the WT mitochondria. The vertical axis corresponds to the significance of individual protein abundance changes, as a –Log_10_ (adjusted *p*-value), with the solid line at a cut off of *p* = 0.05. The horizontal axis displays the Log_2_ (fold change) value. The proteins with increased abundance in the mutant higher than 1.5 (Log_2_ = 0.585) and statistical significance (adjusted *p* < 0.05) are shown in red, and with corresponding decreased abundance in blue. Examples of mitochondrial proteins with more than 4–fold changes are labelled.

A volcano plot of significantly changed protein levels identified in *Δyme1* is shown in Figure 5. In total, 1418 proteins were identified from the WT and *Δyme1* mitochondria samples. The analysis showed that 103 proteins have relative abundances that were significantly increased in the *Δyme1* mitochondria (at least 1.5-fold change, adjusted *p*-value < 0.05), with 480 proteins showing a corresponding significant decrease of at least 1.5-fold. The full list for significantly changed proteins can be found in Supplementary Materials (Supplementary Tables S1 and S2). About 60% (62 proteins) of the proteins with increasing abundance and about ~26% (123 proteins) of proteins with decreased abundance were mitochondria localised. As expected, we found experimentally-confirmed changes in known proteolytic substrate proteins of Yme1 including Ups1 and Ups2 [17], which were significantly increased in abundance. This included seven mitochondrial proteins (Tom70, Mpc2, Ups1, Pth2, Mdm32, Xdj1, Oac1) that have >4-fold increase. In addition to the 62 proteins with increasing abundance, another 24 proteins (including mitochondrial Fmn1, Ict1, Ups2, Cot1) that could only be detected by MS in the *Δyme1* mutant but not the WT strain. Meanwhile, 125 proteins (including Yme1 and 11 other mitochondrial proteins) were detected only in the WT but not in *Δyme1* mitochondria, and 24 mitochondrial proteins showed decreased fold change >4 with some examples indicated in Figure 5.

A functional Gene Ontology analysis revealed a number of mitochondrial processes to be enriched in proteins with increased abundance, including: mitochondria-endoplasmic reticulum (ER) membrane tethering, protein targeting to mitochondrion, cardiolipin metabolic process, protein insertion into mitochondrial membrane, mitochondrial genome maintenance and mitochondrial transmembrane transport (Table 1). For example, Mdm10, Mdm12 and Mmm1, which are components of the endoplasmic reticulum–mitochondrial encounter structure (ERMES) complex involved in connecting the mitochondrial and ER, were increased in abundance in the *Δyme1* mutant. This complex is involved in transporting synthesised membrane lipids from ER to mitochondria and is important for mitochondrial dynamics [30]. Furthermore, the abundance of the cardiolipin biosynthetic process related to proteins Ups1, Ups2, Ict1 and Pgs1 was also up. Interestingly, it was shown that Ups1 and Ups2 antagonistically regulate cardiolipin metabolism in mitochondria [31]. Consequently, this may explain why no significant difference in cardiolipin content was detected in *YME1* deleted yeast cells (Figure 4c,d). Moreover, levels of proteins involved in mitochondrial DNA replication and stability, and mitochondrial fusion and fission, were also increased. This may suggest an increase in mitochondrial dynamics for *Δyme1* mitochondria. Interestingly, Tom6, Tom70 and Tom71, components of the TOM (translocase of outer membrane) complex responsible for protein targeting to mitochondrion, were also identified to be significantly increased (25-fold for Tom70, 3.5-fold for Tom6, 1.5-fold for Tom71) in Yme1 deleted mitochondria. Similarly, Tim17, Tim23 and Tim44, components of the TIM (translocase of inner membrane) complex were identified to be significantly increased (1.8-fold for Tim17, 1.6-fold for Tim23, 1.5-fold for Tim44). These results suggest an increase or change in protein import and mitochondrial protein biosynthesis. Interestingly, Mpc2, a highly conserved subunit of the mitochondrial pyruvate carrier (MPC) mediating import of pyruvate from the cytosol to mitochondria, was significantly increased (19-fold increase) in the mutant mitochondria.

On the other hand, the processes affected by proteins that decrease in abundance are mainly involved in mitochondrial metabolism and energy production, including: aerobic electron transport chain (ETC), TCA cycle (tricarboxylic acid cycle, or Krebs cycle), mitochondrial ATP transmembrane transport, fatty-acyl-CoA metabolic process, pyruvate metabolic process (Table 2). Among these, there were 17 proteins associated with ETC activity, and 13 proteins involved in the TCA cycle, including five overlapping proteins (Sdh1, Shd2, Sdh3, Sdh4, Sdh5) of the succinate dehydrogenase complex.

**Figure 6:**
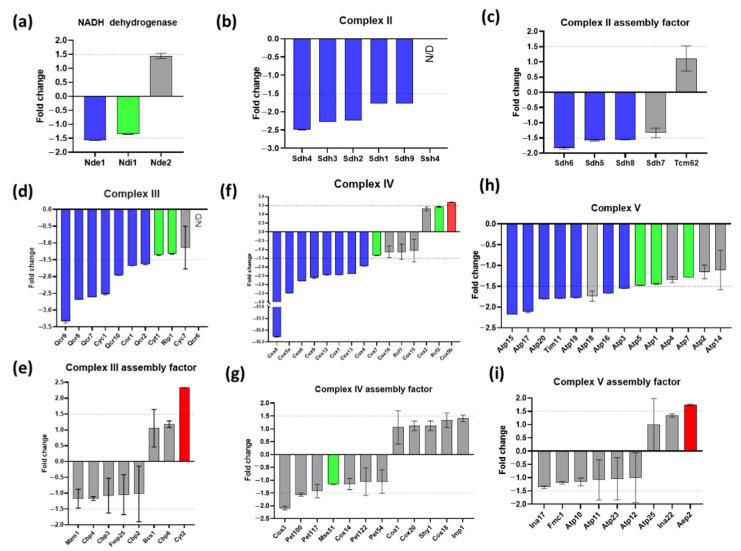
Effect of *YME1* deletion on the relative abundance of constitutive components and assembly factors of various OXPHOS complexes. (**a**) NADH dehydrogenases; (**b**) complex II components, (**c**) complex II assemble factors, (**d**) complex III components, (**e**) complex III assemble factors, (**f**) complex IV components, (**g**) complex IV assemble factors, (**h**) complex V components, (**i**) complex V assemble factors. Blue bars represent proteins with significantly decreased abundance; red bars represent proteins with significantly increased abundance; green bars represent significantly changed proteins (adjusted *p* < 0.05) but with lower fold change then the cut-off point (−1.5 < fold change < 1.5); and grey bars represent non-significant change proteins (−1.5 < fold change < 1.5 and/or *p* > 0.05). N/D: proteins that were not identified in the WT and *Δyme1* samples. Dash lines represent fold change of ±1.5, the cut off for protein abundance change that was considered significantly changed. Error bars represent standard error from MSstats output.

There is no complex I in yeast mitochondria but the three NADH dehydrogenases (Nde1, Nde2, Ndi1) were slightly (Nde1) or not significantly affected (Figure 6a). The effect of *Δyme1* on the individual component of the mitochondrial OXPHOS complexes (II-V) and their assembly factors were analysed and are shown in Figure 6b–i. All complexes II-V had four subunit proteins whose abundances were decreased more than 1.5-fold in *Δyme1* mitochondria (Figure 6b–d,f,h), suggesting that the activity of the complexes II, III, IV and V might be decreased in *Δyme1* mitochondria. On the other hand, the abundances of complex III assembly factor Cyt2 and complex V assembly factor Aep2 were increased in *Δyme1* (Figure 6e,g).

### 2.4. Lost of YME1 Impair the Activities of Mitochondrial OXPHOS Complexes

**Figure 7:**
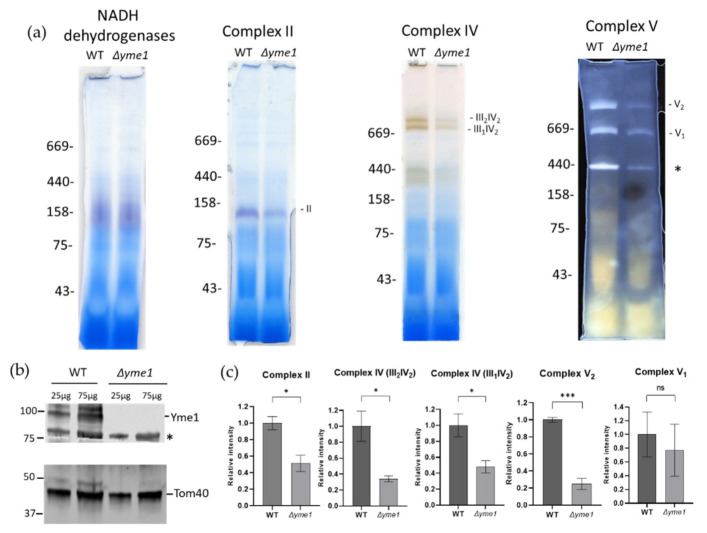
In-gel analysis of effect of *YME1* deletion on mitochondrial OXPHOS complex activities using mitochondria isolated from cells grown in YPGal. (**a**) Blue-native PAGE based in-gel activity assays for mitochondrial NADH dehydrogenases, and complex II, IV and V, as indicated. Mitochondria were isolated from WT and *Δyme1* cells grown in YPGal at 30 °C. In each lane, 50 μg of mitochondria proteins were loaded. Complex II, complex IV in super-complex III_2_IV_1_, III_2_IV_2_ and complex V in monomer (V_1_) and dimer (V_2_) are indicated, and the * indicates an unknown band. (**b**) Western blots with antibodies against mitochondrial Yme1 and Tom40, respectively. WT and *Δyme1* mitochondrial proteins (25 and 75 μg) were separated using reducing SDS-PAGE, then blotted against antibodies for specific protein as indicated. The * indicates a nonspecific band. (**c**) Quantification of the relative activities of the complexes (II, III/IV, V) as shown in A. The error bars represent SEM of three independent experiments with the same mitochondrial preparation (n = 3). The statistic was done using Student’s *t* test, ns *p* > 0.05; * *p* < 0.05;; *** *p* < 0.001.

To verify the proteomics results and investigate the effects of *YME1* deletion on mitochondrial OXPHOS complexes in more detail, the activities of complexes II and V, and the III/IV super-complex, were assessed using BN-PAGE coupled with in-gel activity-staining assays as described previously [32]. An equal amount of the WT and mutant mitochondrial proteins were separated by BN-PAGE, followed by incubation with a reaction buffer specific for the activity of complex II, V, or complex IV in the III/IV super-complexes, respectively (Figure 7a). The intensities of activity-staining were quantified (Figure 7b), showing that *Δyme1* had significantly reduced activities of complex II and complex V dimer (V_2_), but no significant difference for monomeric complex V (V_1_). In yeast, the complexes III and IV form two super-complexes: III_2_IV_1_ and III_2_IV_2_ [33]. The relative activity of complex IV in these super-complexes decreased to about 40% that of the WT in the *Δyme1* mutant (Figure 7b). SDS-PAGE and Western blotting with antibodies against Yme1 and mitochondrial marker protein Tom40 confirmed *YME1* deletion and equal sample loading (Figure 7c).

**Figure 8:**
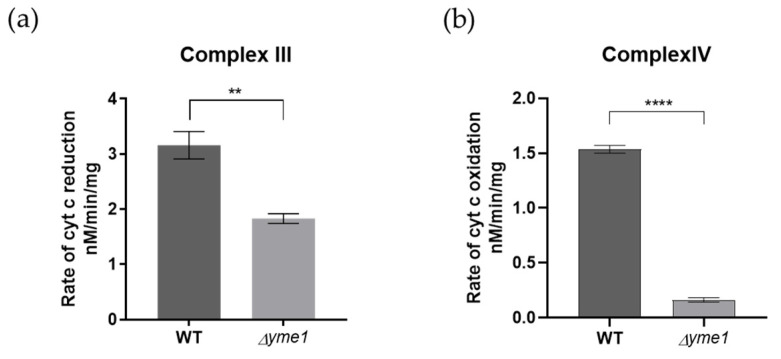
In-solution activity analysis of the mitochondrial complexes III and IV. Mitochondria were isolated from cells grown in YPGal at 30 °C. (**a**) The activity of complex III, and (**b**) complex IV was measured using spectroscopic method. The error bars represent SEM of three independent experiments with the same mitochondrial preparation (n = 3). The statistic was done using Student’s *t* test, ** *p* < 0.01; **** *p* < 0.0001.

Next, to decipher the effect of Yme1 deletion on the complex III/IV activity further, the relative activities of individual complexes III and IV were analysed using solution-based biochemical assays as described previously [34]. The results showed that both complex III and IV activities were significantly reduced in *Δyme1* (Figure 8). They were decreased to about 58% for the complex III and 13% for the complex IV, in agreement with the proteomics results that indicated the abundance of the complex IV subunits was affected most, which decreased more than that of the complex III subunits (Figure 7). In summary, the results of biochemical activity analyses were consistent with our mass spectrometry proteomics results, confirming that *Δyme1* has an adverse effect on the activity of OXPHOS complexes II–V.

## 3. Discussion

Yme1 is a multifunctional protein, involved in mitochondrial protein import, folding, maturation and degradation. Although Yme1 has been shown to play an important role in maintaining the electron transport chain (ETC) function, specific details are lacking. In this study, we investigated how *YME1* deletion affects the chronological life span of yeast and mitochondrial protein homeostasis, as well as the function of OXPHOS complexes under fermentative growth conditions. Our results showed that whilst there are obvious consequences of *YME1* deletion on normal cell growth under fermentative conditions, the chronological life span is significantly affected, suggesting that Yme1 is required for longevity. Hence, a gradual and accumulated effect on mitochondrial function during fermentative growth may account for the shortened chronological life span. This is consistent with other studies that have shown a decline of mitochondrial function is linked to aging [35,36,37].

Previous studies showed that unassembled Cox2 (the subunit II of cytochrome oxidase) can be degraded by Yme1 [12,14,38]. Other studies demonstrated that Yme1 plays a chaperone role in the folding and/or assembly of Cox2 [19,39]. Here, we examined how *YME1* deletion affects the activity of all the complexes of OXPHOS and revealed that they are all decreased in relative abundance. Among these, the activity of complex IV is most affected by *YME1* deletion, with its activity only about 10% that of the WT (Figure 7b), whilst activity of the III/IV super-complex only decreased to about 40% that of the WT (Figure 6b). Furthermore, our proteomics analysis showed that the abundances of several components of the complex II (Sdh1–4, Sdh9), III (Cyc1, Cor1, Qcr2 and Qcr7–10), IV (Cox1, Cox4, Cox5A, Cox6c Cox9, Cox12 and Cox13) and V (Atp3, Atp15, Atp16, Atp17, Atp19 and Atp20) were decreased, with subunits of complex IV Cox5a, Cox6 and Cox8 being most affected (Figure 6). Thus, these results suggest that *YME1* deletion itself has no or little effect on the assembly of the III/IV super-complex, but the activity decrease is most likely due to the abundance decrease of some subunits of the complex IV. In this study, the abundance of Cox2 was found to be increased by 1.3-fold, albeit statistically not significant (*p* > 0.05), which indicates that *YME1* deletion may lead to a slight accumulation of unassembled Cox2.

Cardiolipin has been shown to be important for maintaining optimal activity of ETC complexes and structural organisation of respiratory complexes [40,41,42]. Moreover, formation of the ETC super-complex III_2_IV_1_ and III_2_IV_2_ is dependent in preference of cardiolipin [43]. Interestingly, the cardiolipin content and membrane potential were not obviously affected by *YME1* deletion, though both Ups1 and Ups2, known regulators of cardiolipin and substrates of Yme1, were increased in abundance. The lack of effect on cardiolipin may be the result of the opposing effects of Ups1 and Ups2 in regulation of cardiolipin metabolism compensating for each other [31]. This is consistent with the above conclusion that the assembly of the III/IV super-complexes is not affected by *YME1* deletion.

Another interesting finding of our mitochondrial proteomics analysis was that the level of Mpc2, an important component of mitochondrial pyruvate carrier (MPC), was significantly (19-fold) increased (Figure 4). In yeast, MPC consists of Mpc1 and Mpc2 during fermentative growth, or Mcp1p and Mpc3p during respiratory growth. MPC plays a key role in bridging the cytosolic glycolysis and mitochondrial TCA cycle by mediating pyruvate uptake from the cytosol to the mitochondrial matrix. Our mitochondria proteomics analysis showed that whilst Mpc2 and Mpc3 were about 19.5-fold and 2.5-fold increase in abundance under our experimental conditions, no significant change was observed for Mpc1. Consistent with our conclusion, the Y3k yeast whole cell proteomic analysis [23] also revealed that Mpc2 was up-regulated in Yme1 deleted yeast cells but not Mpc1 (http://y3kproject.org/projectInfo.php (accessed on 16 September 2022)). Whist the Mpc1/Mpc2 heterodimer is functional in pyruvate import, Mpc2 can also form a homodimer, but its function is unknown. Thus, Yme1 may be involved in mitochondrial biogenesis of Mpc2 and Mpc3, or these proteins may be proteolytic substrates of Yme1, which remains to be addressed. Moreover, Yme1 may play a role in regulating the function of the TCA cycle through regulation of the biogenesis and/or function of Mpc2. It will be interesting and important to understand how Yme1 is involved in regulating the homeostasis and function of MPC and TCA cycle in future studies.

Taken together, this report showed that Yme1 plays an important role in maintaining mitochondrial protein homeostasis and the chronological life span of yeast under both respiratory and fermentative growth conditions. *YME1* deletion impairs the activity of all four (II-V) complexes of the yeast OXPHOS chain even under fermentative conditions. Furthermore, our results suggested that Yme1 also plays a role in maintaining pyruvate metabolism and the TCA cycle, which requires further experimental studies to verify and understand the molecular mechanisms in detail.

## 4. Materials and Methods

### 4.1. Yeast Strains

The yeast strains used in this study are *S cerevisiae* BY4741 WT (*MATa his3Δ1 leu2Δ0 met15Δ0 ura3Δ0)* and BY4741 *YME1* deletion (*MATa his3Δ1 leu2Δ0 met15Δ0 ura3Δ0 Δyme1::kanMX4*) and are gifted from Professor Daniela Delneri, University of Manchester.

### 4.2. Media and Growth Conditions

To monitor culture growth, cells were first grown in YPD (2% *w*/*v* glucose; 2% *w*/*v* Peptone; 1% yeast extract), YPGal (2% *w*/*v* galactose; 2% *w*/*v* Peptone; 1% yeast extract) or YPEG (2% *v*/*v* ethanol; 2% *w*/*v* glycerol; 2% *w*/*v* Peptone; 1% yeast extract) media to stationary phase for 48 h at 30 °C. Cells were then inoculated to a starting optical density of 0.03 in respective media into a 96-well clear flat bottom plate. OD_600_ were monitored for 48 h at 30 °C with shaking at 200 rpm using an infinite F200 Pro plate reader (Tecan, Männedorf, Switzerland.

### 4.3. Spot Tests

Cells were grown in YPD, YPGal or YPEG to the exponential phase (OD_600_ = 0.5) at 30 °C. Cells were then harvested and resuspended in 1x PBS and adjusted to OD_600_ of 10^−1^, 10^−2^, 10^−3^, 10^−4^, 10^−5^ before spotting onto fresh YPGal or YPEG plates. Plates were incubated at 30 for 2 days before digital pictures were taken. The experiment was repeated three times.

### 4.4. Cardiolipin Content and Membrane Potential Analyses

Cells grown in YPGal were collected at exponential or stationary growth phases. Cells equivalent to OD_600_ = 1 were incubated with 100 nM of 10-N-nonyl acridine orange (NAO) for cardiolipin content, or 40 nM of DiOC_6_ in 1x PBS for membrane potential respectively, at 30 °C for 30 min. After incubation, the cells were washed with 1x PBS twice prior to analysis. For both cardiolipin and membrane potential analysis, samples were submitted to a BD LSRFortessa^TM^ cell analyser (Becton, Dickson and Company, Franklin Lakes, NJ, United States) and analysed with the blue laser set at excitation wavelength 488 nm and emission wavelength 530 nm with a 30 nm bandpass filter. For each sample, 10,000 cells were recorded. Raw outputs were processed by inbuilt software BD FACSDiva^TM^ (Version 9, Becton, Dickson and Company, Franklin Lakes, NJ, United States). Statistical analysis was performed using Prism 7.0 with Student’s t test. For this assay, three independent measurements were performed with at least three cell cultures each.

### 4.5. Yeast Chronological Life Span (CLS) Analysis

For the colony forming unit (CFU) count assay, stationary phase yeast precultures were used to inoculate fresh YPGal or YPEG media at starting OD_600_ of 0.05 and grown at 30 °C for 2 days to reach the stationary phase. After reaching the stationary phase (day 0), OD_600_ = 0.1 worth of cell suspension was spread on respective media plates every 4 days and incubated at 30 °C for 2 days prior to colony forming unit count.

For the fluorescent dye staining coupled flow cytometry method, 50 mL of fresh SCD+ (2% *w*/*v* glucose, 0.17% yeast nitrogen base without amino acids, 1x Kaiser amino acid mixes supplemented with 4-fold excess of uracil, leucine, tryptophan, adenine and histidine) media were inoculated to a starting OD_600_ of 0.25 and grown at 30 °C with shaking at 200 rpm. Subsequently, samples were taken every 3 days for cell viability assay by flow cytometry analysis following methods adapted from Ocampo and Barrientos (2011). Then, 50 µL of cell culture were mixed with 200 µM propidium iodide in 1× PBS to a final volume of 1 mL. Samples were incubated at 30 °C for 30 min prior flow cytometry analysis using LSRFortessaTM cell analyser (Becton, Dickson and Company, Franklin Lakes, NJ, United States). The cell analyser was set with a blue laser with an excitation wavelength at 561 nm and a yellow laser with an emission wavelength at 610 with 20 nm bandpass filter at a voltage of 500 V. For this assay, three independent measurements were performed with at least three cell cultures each.

### 4.6. Mitochondria Isolation

Mitochondria were isolated by fractionation as described by Gregg et al. (2009) [44]. Briefly, cells were grown in YPGal and harvested at late exponential phases of OD ~1.5. After pre-treatment with DTT buffer (100 mM Tris-H_2_SO_4_ pH 9.4, 10 mM DTT) at 30 °C for 20 min, the cell wall was digested with zymolyase 20T (Seikagaku Corporation, Tokyo, Japan) in zymolyase buffer (20 mM potassium phosphate buffer pH 7.4, 1.2 M sorbitol) for 1 h at 30° with shaking at 70 rpm. The resulting spheroplasts were checked under a light microscope to confirm cell wall digestion. Then, the spheroplasts were lysed in homogenisation buffer (20 mM HEPES-KOH pH7.4, 0.6 M sorbitol, 1 mM phenylmethylsulfonyl fluoride (PMSF)) on-ice using a glass Teflon homogeniser. The resulting spheroplasts were subjected to two centrifugation steps at 1500× *g* to remove the cell debris and nuclei. Crude mitochondrial materials were isolated from the supernatant by centrifugation at 12,000× *g* at 4 °C for 10 min, then resuspended in SEM buffer (250 mM sucrose, 0.6 M Sorbitol, 20 mM HEPES-KOH pH 7.4). Protein concentrations were checked by measuring absorbance at 280 nm, assuming an absorbance value of A_280_ = 0.21 would equate to 10 mg/mL of crude mitochondrial extract. After adjusting to a final concentration of 10 mg/mL, the crude mitochondria were then flash frozen and stored at −80 °C.

For mass spectrometry proteomics analysis, four 1 L cell cultures for each strain (WT and *Δyme1*) were prepared in parallel for mitochondrial isolation as explained above. The freshly prepared crude mitochondria were further enriched using sucrose gradient centrifugation. Sucrose gradients were prepared in a Beckman Ultra-Clear centrifuge tube (Beckman Coulter, Brea, CA, United States) as follow: 1.5 mL of ice-cold 60% (*w*/*v*) sucrose in homogenisation buffer, 4 mL of 32% sucrose in homogenisation buffer, 1.5 mL of 23% (*w*/*v*) sucrose in homogenisation buffer and 1.5 mL of 15% (*w*/*v*) sucrose in homogenisation buffer. Then, 3 mL of crude mitochondrial extract was layered on top of the 15% (*w*/*v*) sucrose and centrifuged in a SW41 Ti swinging-bucket rotor (Beckman Coulter, Brea, CA, United States) for 1 h at 134,000× *g* at 4 °C. The sucrose gradient enriched mitochondria were collected at the interface between 60% and 32% sucrose and washed with SBB7.4 buffer prior to storage at −80 °C. The WT and *Δyme1* enriched mitochondria samples (four of each) were prepared for proteomics analysis.

### 4.7. Blue Native in-gel Respiratory Complex Activity Assays

Crude mitochondrial extracts were prepared by homogenised in solubilisation buffer (50 mM NaCl, 50 mM Imidazole-HCl pH 7, 2 mM 6-Aminohexanoic acid, 1 mM EDTA), followed by solubilisation by adding Digitonin at 3 g/g and incubated for 10 min on-ice, and then centrifugation at 20,000× *g* for 20 min. Resulting supernatants were collected. Glycerol was added to a final concentration of 5% and Coomassie blue suspension (5% *w*/*v* in 500 mM 6-aminohexanoic acid) was added to give a detergent/dye ratio of 8 g/g.

For activity assays, the crude mitochondrial protein extract was first resolved using 4–16% bis-tris blue native PAGE (Catalog number BN1002BOX, Invitrogen, Waltham, MA, United States) with a loading of typically 50 µg per lane. A line of the resulting gel was cut and incubated in 20 mL of a specific respiratory complex reaction buffer. For NADH dehydrogenase activity assay, the resulting gel was incubated in 50 mM Tris-HCl pH7.4, 1 mg/mL NADH and 2 mg/mL NBT until a darken band could be observed. For the complex II activity assay, the resulting gel was incubated in 50 mM KH_2_PO_4_ pH7.4, 84 mM succinate, 0.2 mM PMS and 2 mg/mL NBT until a dark band at position ~140 kDa could be observed. For the complex IV activity assay, the gel was incubated in 10 mM KH_2_PO_4_ pH7.4, 1 mg/mL DAB and 1 mg/mL cytochrome c. The gel was incubated until yellow bands at a position higher than ~669 kDa could be observed. For the complex V activity assay, the gel was incubated in 35 mM Tris-HCl pH7.4, 270 mM glycine, 14 mM MgSO_4_, 0.2% *w*/*v* Pb(NO_3_)_2_, 8 mM ATP until two white bands at position 600 kDa and 1250 kDa could be observed. After incubation, all reactions were stopped by transferring and incubating the gel into fixing solution (40% *v*/*v* methanol, 10% *v*/*v* acetic acid). Gels were then scanned using Fujifilm LAS-1000(Fujifilm, Tokyo, Japan) and the digitised pictures were analysed using densitometry using Image J (version 1.50b, https://imagej.nih.gov/ij/index.html (accessed on 16 September 2022)). Three independent measurements were performed with the same mitochondrial preparation.

### 4.8. The Complexes III and IV Activity in-solution Assays

The activity of mitochondrial complexes III and IV in solution was analysed as described in [34]. Three independent measurements were performed with the same mitochondrial preparation. The complexes III activity was determined by measuring the rate of reduction of cytochrome c by ubiquinol at 550 nm at 30 °C. Then, 10 µg of crude mitochondrial protein extract was added to pre-heated (30 °C) complex III reaction mixture (50 mM potassium phosphate buffer pH7.4, 2 mM NaN_3_, 50 µM cytochrome c, in the presence or absence of 10 µg/mL antimycin A). Absorbance at 550 nm was monitored for 2 min and the activity was calculated based on the increase in absorbance at 550 nm by the following equation:

[(ΔA_550nm_^−Antimycin^ − ΔA_550nm_^+antimycin^) ∗ reaction volume (cm^3^)]/[ε_1_ ∗ light path (cm) ∗ protein (mg)] (1)

ε_1_ = 21 mM^−1^cm^−2^.

The complex IV activity was determined by measuring the rate of oxidation of reduced cytochrome c. Then, 10 µg of crude mitochondrial extract was mixed with complex IV reaction mixture (10 mM phosphate buffer pH 7.4, 8 mM reduced cytochrome c, in the presence or absence of 2 mM KCN). Absorbance at 550 nm was monitored for 2 min and the complex activity was calculated based on decrease in absorbance change at 550 nm.

[ΔA_550_^−KCN^ − ΔA_550_^+KCN^ * reaction volume (cm^3^)]/[ε_1_ ∗ light path (cm) ∗ protein (mg)] (2)

ε_1_ = 21 nM^−1^cm^−2^.

### 4.9. Mass Spectrometry Analysis

For the mass spectrometry analysis, a protocol from the mass spectrometry facility in the Faculty of Biology, Medicine and Health of the University of Manchester was used. Briefly, 200 μg samples of purified mitochondrial extract were first incubated with sample buffer (5 mM DTT, 50 mM TEAB pH 7.5, 5% SDS) for 10 min at 60 °C, followed by alkylation by addition of 15 mM IAM and incubated in the dark, at room temperature for 30 min. Then, 5 mM of DTT were added to quench the alkylation reaction. Samples were then centrifuged at 14,000× *g* for 10 min, and the resulting pellets were collected as protein lysate. Afterwards, the lysates were mixed with 1.2% aqueous phosphoric acid, followed by S-trap binding buffer (100 mM TEAB (pH7.1) in 90% methanol), then loaded onto a 96-well S-Trap plate (ProtiFi). Samples were digested by incubation with trypsin in digestion buffer (50 mM triethylammonium bicarbonate) at a ratio of 1:10 (enzyme:protein) for 1 h at 47 °C. Digested peptides were eluted by 5% aqueous acetonitrile containing 0.1% aqueous formic acid. To desalt eluted peptides, samples were loaded onto FiltrEX desalt filter plate (Corning, New York, United States) containing POROS R3 beads and washed with 0.1% aqueous formic acid twice. Samples were then eluted with 0.1% aqueous formic acid containing 30% acetonitrile. Collected elusions were dried to completeness in a Heto vacuum centrifuge then stored at 4 °C.

Peptide samples were analysed by the faculty mass spectrometry core facility. Briefly, samples were separated using a multistep gradient from 95% buffer A (0.1% FA in water) and 5% buffer B (0.1% FA in acetonitrile) to 7% buffer B at 1 min, 18% buffer B at 35 min, 27% buffer B at 43 min and 60% buffer B at 44 min at 300 nL min^−1^ with a 75 mm × 250 μm i.d. 1.7 μM CSH C18 analytical column (Waters, Wilmslow, UK). Peptides were selected for fragmentation automatically by data dependent analysis.

The raw data from mass spectrometry were processed with MaxQuant [26], using software version 1.6.5.0. Search parameters were set as follows: label free experiment with default settings; cleaving enzyme trypsin with two missed cleavages; Orbitrap instrument with default parameters; variable modifications: oxidation (M) and Acetyl (protein N-term); first search as default; in global parameters, the software was directed to the S288C reference FASTA file ‘orf_trans.fasta’ from the *Saccharomyces* genome database (SGD) (accessed 22 October 2022) [45]; for advanced identification “Match between runs” was checked; for protein quantification only used unique, unmodified peptides. All other MaxQuant settings were kept as default. The false discovery rate (FDR) for both accepted peptide spectrum matches and protein matches were set to 1%, using default parameters. Differential expression calculations were performed using the MSstat package (version 3.14.1) in R (version 3.5.2) [27] to identify and quantify proteins that display significantly changes in abundance, either up or down. Proteins displaying significant changes in the mutant strain were investigated for significant functional enrichment using the SGD gene ontology knowledgebase and Gene Ontology Resource (http://www.geneontology.org (accessed on 16 September 2022)) [28,29]. Components of OXPHOS complexes were assigned based on the previous study by [24].

## Figures and Tables

**Table 1:** Mitochondrial processes affected by proteins that increase in abundance in *yme1* mutant mitochondria.

Mitochondrial Biological Process	Proteins Involved	FDR-Corrected *p*-Value
Mitochondrion-ER membrane tethering (GO:1990456)	Mdm10, Mdm12, Mmm1	2.4 × 10^–2^
Cardiolipin metabolic process (GO:0032048)	Ict1, Pgs1, Ups1, Ups2	2.0 × 10^–2^
Protein insertion into mitochondrial membrane (GO:0051204)	Mdm10, Mdm12, Mmm1, Tom70, Tom71, Sam37	2.0 × 10^–3^
Mitochondrial genome maintenance (GO:0000002)	Ilv5, Mdm10, Mdm12, Mdv1, Mgm1, Mip1, Mmm1, Rrg8, Tim17	1.4 × 10^–4^
Mitochondrial transmembrane transport (GO:1990542)	Fsf1, Mpc2, Mpc3, Tom70, Tom71, Xdj1, Tim23, Tim17, Tom6, Tim44	1.6 × 10^–4^

**Table 2:** Mitochondrial processes affected by proteins that significantly decrease in abundance in *yme1* mutant mitochondria.

Mitochondrial Biological Process	Proteins Involved	FDR-Corrected *p*-Value
Aerobic electron transport chain (ETC) (GO:0019646)	Cor1, Cox13, Cox4, Cox5a, Cox6, Cox8, Cox9, Cyc1, Qcr10, Qcr2, Qcr7, Qcr8, Qcr9, Sdh1, Sdh2*, Sdh3, Sdh4, Sdh5	4.85 × 10^–6^
TCA cycle (GO:0006099)	Aco1, Cit1, Fum1, Idh1, Idh2, Kgd1, Mdh1, Mdh3, Sdh1, Sdh2, Sdh3, Sdh4, Sdh5	1.0 × 10^–4^
Mitochondrial ATP transmembrane transport (GO:1990544)	Aac1, Odc1, Odc2, Pet9	4.2 × 10^–2^
Fatty-acyl-CoA metabolic process (GO:0035337)	Faa1, Faa3, Faa4	4.2 × 10^–2^
Pyruvate metabolic process (GO:0006090)	Cdc19, Eno2, Glk1, Gpd1, Gpm1, Hxk1, Hxk2, Pdc1, Pfk1, Pfk2, Pgk1, Pyc1, Tdh3	5.6 × 10^–4^

* Sdh2 is a subunit of the succinate dehydrogenase complex involved in the electron transport chain, but at the time of the writing Sdh2 was not annotated in the GO term aerobic electron transport chain (GO:0019646).

## Data Availability

The mass spectrometry proteomics data have been deposited to the ProteomeXchange Consortium via the PRIDE [46] partner repository with the dataset identifier PXD036367.

## Supplementary Materials

The following supporting information can be downloaded at: https://www.mdpi.com/article/10.3390/ijms232213694/s1.
